# Adiposity Is a Key Correlate of Circulating Fibroblast Growth Factor-21 Levels in African Males with or without Type 2 Diabetes Mellitus

**DOI:** 10.1155/2018/7461903

**Published:** 2018-09-12

**Authors:** Fredirick L. Mashili, Kaushik Ramaiya, Janet Lutale, Marina Njelekela, Filbert Francis, Juleen Zierath, Anna Krook

**Affiliations:** ^1^Department of Physiology, Muhimbili University of Health and Allied Sciences, Box 65001, Dar es Salaam, Tanzania; ^2^Tanzania Diabetes Association, Box 65201, Dar Es Salaam, Tanzania; ^3^National Institute of Medical Research, Box 5004, Tanga, Tanzania; ^4^Department of Physiology and Pharmacology, SE-17177 Stockholm, Sweden

## Abstract

**Background:**

Fibroblast growth factor-21 is an endocrine regulator with therapeutic and diagnostic potential. The levels and pattern of circulating FGF-21 have been described mainly in European and Asian populations. Given its strong association with adiposity, and the reported ethnic variabilities in body composition, examining FGF-21 in an African population is crucial.

**Methods:**

We measured levels of circulating FGF-21 in 207 overweight and obese Tanzanian males with or without type 2 diabetes mellitus (T2DM), and using statistical approaches, we explored their relationship with anthropometric and biochemical parameters.

**Results:**

Consistent with previous reports from European and Asian populations, we found higher levels of FGF-21 in people with T2DM compared to those without the disease. Based on statistical models, measures of adiposity explained up to 59% of the variability in FGF-21 levels in the circulation.

**Conclusion:**

Levels of circulating FGF-21 in overweight and obese African males are higher in T2DM and strongly correlate with measures of adiposity.

## 1. Introduction

Fibroblast growth factor-21 is a circulating protein with a variety of metabolic effects. FGF-21 can stimulate glucose uptake, lower blood glucose and lipid levels, and ameliorate several metabolic perturbations in obesity and type 2 diabetes mellitus (T2DM) [1]. The initial discovery of FGF-21 metabolic function came from experiments done in 3T3-L1 adipocytes, demonstrating its ability to facilitate cellular glucose uptake independent of insulin [2]. Subsequent exploration both in humans and animal models revealed a number of positive biological effects including weight loss and blood lipid lowering [2–6]. Based on that, FGF-21 has received significant scientific attention over the past decades. Due to the positive biological effects on metabolism, FGF-21 has been suggested as a future therapeutic agent against T2DM, dyslipidemia, and atherosclerosis [2–6]. In addition, circulating FGF-21 levels at baseline have been shown to predict the development of metabolic diseases in different populations [7–9], indicating that FGF-21 can also be used as a screening and diagnostic biomarker for metabolic diseases [10].

Despite the therapeutic promises, results from studies conducted in different populations have revealed intriguing relationship between FGF-21 circulating levels and variety of pathophysiological and disease states [7, 11]. Unexpectedly, levels of circulating FGF-21 have been found to be higher in people with obesity, T2DM, and NAFLD, when compared to normal individuals [7, 11]. Given its positive metabolic effects, FGF-21 levels were expected to be higher in normal than in individuals with perturbed metabolism. For that reason, circulating FGF-21 levels display a paradoxical elevation in obesity-related diseases [7, 11]. This has led to speculations that obesity and related conditions could be FGF-21 resistant states.

Presence of FGF-21 resistance in obesity has indeed been demonstrated in animal models [12]. To further explore the paradox, many studies have examined FGF-21 serum levels in people with varying degrees of glucose tolerance and adiposity [13–15]. Compared to people with normal glucose, tolerance FGF-21 levels in the circulation are consistently higher in T2DM and show a continuous increase with worsening glucose tolerance (low in NGT, intermediate in IGT, and higher in T2DM) [13, 14]. Similarly, studies conducted in different populations and ethnicities have found higher levels of FGF-21 in serum of people with conditions of high adiposity such as NAFLD [7, 11, 16].

A growing body of evidence points to visceral, specifically liver fat content as a key correlate and determinant of circulating FGF-21 levels both in animals and humans [16–22]. This implies that levels of FGF-21 in the circulation are likely related to body fatness (adiposity) and not other aspects of body composition. Body composition is traditionally divided into two main parts, lean body mass (LBM) and fat mass (FM) [23]. Scott *et al* demonstrated a significant variability in body adiposity at a given body size across ethnicities. In their study, South Asians were found to have a phenotype of higher FM and lower LBM compared to other ethnic groups. This high adiposity accounted for greater levels of insulin and insulin resistance that was observed among South Asians [24]. Several other studies have demonstrated ethnic and regional variabilities in adiposity that also accounted for significant differences in metabolic profiles across ethnic groups [25–29]. Collectively, these reports provide evidence for ethnic and regional differences in adiposity and metabolic profile.

Studies comparing metabolic profiles between African and Caucasians have reported differences in the levels of adipocytokines between the two populations. At a similar BMI, Africans living in Tanzania had 50% higher leptin levels when compared to Caucasians living in Sweden [30]. Given the ethnic and regional variations in body adiposity and metabolic profile [31–35], and the recently reported gender differences in circulating FGF-21 levels [36], it is logical to speculate that the status and pattern of circulating FGF-21 could be different in an African population. While FGF-21 has been extensively explored in European and Asian populations, data from Sub Saharan Africa (SSA) are lacking. To the best of our knowledge, this is the first study to describe levels and pattern of circulating FGF-21 in a selected population of overweight and obese African males.

To address patterns of circulating FGF-21, we determined FGF-21 in a selected population from SSA. Levels of circulating FGF-21 were measured in overweight and obese African males, with or without T2DM, and relationship with anthropometric and biochemical parameters was subsequently examined.

## 2. Subjects and Methods

### 2.1. Subjects

Study participants were males with or without type 2 diabetes mellitus (T2DM), who were recruited from the community and different diabetic clinics in Dar es Salaam. Participants were recruited through adverts posted in different hospitals and several gathering sites within the communities in all the three districts of Dar es Salaam region. After providing written informed consent, all participants were instructed to report at the Muhimbili National Hospital diabetic clinic where all the assessments took place. Diabetic and nondiabetic subjects who were 30 years and above were matched for BMI. Subjects with known type 1 diabetes mellitus (T1DM), type 2 diabetes patients on insulin and any diabetic patient with diabetes duration of more than one year were excluded. Also, patients with known complications such as cardiovascular and renal diseases were excluded. The Muhimblili University of Health and Allied Science (MUHAS) institutional review board evaluated and approved the study protocol.

### 2.2. Clinical and Biochemical Assessments

Subjects reported at the MNH diabetic clinic for assessments after an overnight fast (at least 8 hours). Demographic, dietary, and other behavioral information were obtained using standardized questionnaires. Global physical activity questionnaire was used to assess levels of physical activity. Systolic and diastolic blood pressures were measured three times using calibrated digital sphygmomanometer (Omron). Fasting venous blood was drawn and processed for measurements of plasma glucose, insulin levels, lipids, and glycated hemoglobin. Circulating FGF-21 was also measured in serum using commercial ELISA kits (Bio Vendor Laboratory Medicine, Inc., Modrice, Czech Republic) according to manufacturer's instructions. FGF-21 assay was performed as previously described [11, 37].

### 2.3. Anthropometric Assessments

Weight, height, and waist circumference were measured using standardized instruments and protocols, as previously described [38]. BMI was calculated using the formula weight (kg)/Height^2^ (m^2^). BMI cut off for overweight and obesity were defined according to WHO standards [39].

### 2.4. Assessment of Body Composition

Body composition was assessed by bioelectric impedes analysis (BIA) using a calibrated Tanita BC-418MA Segmental Body Composition Analyzer (Tanita Corporation). All the measurements were done in light clothing and bare feet. An adjustment of 0.8 kg was done to account for clothing. The BIA measurement protocol was according to manufacturer's guidelines, and all the measurements were done at the frequency of 50 HZ. The inbuilt equation was used to automatically estimate total body water (TBW), fat-free mass (FFM), body fat (BF), and percentage body fat (%BF).

### 2.5. Statistical Analysis

Data are presented as mean ± standard deviation. The statistical analysis was performed using STATA version 12.0. Differences in levels of circulating FGF-21 between nondiabetic and type 2 diabetes subjects were compared using an unpaired *t*-test. Guided by results of normal distribution tests, regression analyses were performed using Pearson's or Spearman rank correlation test and were used to evaluate the relationships between circulating FGF-21 levels and other clinical parameters (anthropometric and biochemical). Finally, a multiple stepwise regression analysis was used to evaluate the independent determinants of circulating FGF-21 levels.

## 3. Results

### 3.1. Circulating FGF-21 Levels Are Elevated in African Males with T2DM

Clinical and biochemical characteristics of the study participants are shown in Table 1. The two groups (Nondiabetic vs T2DM subjects) were matched for BMI. Patients with T2DM were significantly older compared to nondiabetic subjects (Table 1). Waist circumference, body fat percentage, fasting plasma glucose, systolic blood pressure, fasting triglyceride, and HbA1C were higher in T2DM patients compared with nondiabetic subjects (Table 1). The level of HDL cholesterol was lower in T2DM patients (Table 1). Circulating FGF-21 levels were significantly higher in T2DM patients (Table 1).

### 3.2. Relationship between Circulating FGF-21 Levels and Clinical Parameters

**Figure 1:**
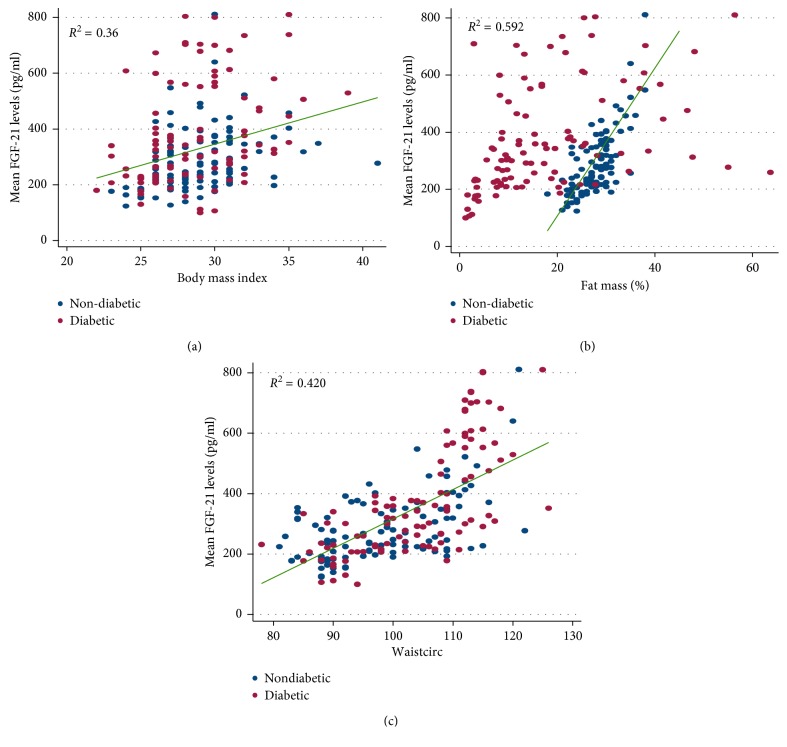
Correlates of circulating FGF-21 levels in nondiabetic (•) and T2DM patients (o) (*n*=106 and 101 in nondiabetic and T2DM patients, respectively). Correlation with (a) BMI, (b) percentage body fat, and (c) waist circumference.

A Spearman rank correlation analysis was performed to explore the relationship between circulating FGF-21 levels and a number of anthropometric and biochemical parameters (Table 2). The analysis was done as a combined group of nondiabetic and T2DM subjects and also in individual groups (nondiabetic and T2DM subjects separately). Measures of adiposity (BMI, waist circumference, and body fat percentage) were highly correlated with circulating FGF-21 levels while measures of glucose control (HbA1C) only showed a weak correlation. In a combined analysis, BMI, WC, and body fat percentage explained 36%, 42%, and 59% of the variations in circulating FGF-21 levels, respectively (Figure 1). Combined and individual groups analysis did not show any marked difference.

### 3.3. Independent Determinants of Circulating FGF-21 Levels

Multiple stepwise regression analysis was performed to further explore the factors associated with FGF-21 circulating levels and identify its independent determinants (Table 3). In this analysis, waist circumference and body fat percentage were identified as strong independent determinants of circulating FGF-21 levels in this population (Table 3).

## 4. Discussions

This study aimed to substantiate the previously reported pattern of circulating FGF-21 in an African population. Here, we report a strong association between circulating FGF-21 levels and measures of adiposity in overweight and obese African males, with or without T2DM. Our findings indicate that FGF-21 biological potentials observed in Caucasian and Asian populations could also apply in African populations. To the best of our knowledge, FGF-21 has not been explored in African populations, and these results expand our understanding of FGF-21 regulation in overweight and obese African males.

Several studies have reported a significant association between adiposity and levels of circulating FGF-21 in different populations [16–22]. In our study population, BMI, waist circumference (WC), and percentage body fat, which are surrogate measures of adiposity, were significantly associated with levels of circulating FGF-21. Similar association was observed when nondiabetic and type 2 diabetic subjects were analyzed in combined and separate groups. In a combined group, measures of adiposity explained up to 59% of the variability in the levels of FGF-21 in the circulation, implying that adiposity plays a key role in the regulation of FGF-21 in the circulation. In addition, these results show that, in a state of increased weight (overweight and obesity), T2DM does not expressively alter the biological relationship between adiposity and circulating FGF-21.

We observed similar pattern of relationship between circulating FGF-21 and measures of adiposity in a Swedish cohort [37]. However, in our Swedish study, FGF-21 correlated with BMI in T2DM but not in nondiabetic subjects [37]. This is contrary to the current findings where the association was maintained even in nondiabetic subjects. While our Swedish study included both males and females, the current analysis only involved males. Gender dimorphism in the relationship between circulating FGF-21 and cardiometabolic parameters has been previously reported [40] and could partly explain the discrepancy observed in the two studies. Furthermore, unlike the Swedish cohort, the current study did not include normal weight individuals. Since obesity has been identified as a state of FGF-21 resistance [12], it is logical to speculate that the higher levels of circulating FGF-21 are partly driven by high adiposity that accompanies overweight and/or obesity. The association between adiposity and circulating FGF-21 levels is therefore much stronger in conditions of high adiposity (overweight and obesity). For that reason, the inclusion of normal weight individuals in our Swedish population could have diminished the strength of association between circulating FGF-21 and BMI in nondiabetic subjects.

The interplay between adiposity and circulating FGF-21 is closely linked to visceral fat content [16–22], and the underlying mechanisms have been extensively explored [41–45]. FGF-21 is secreted by adipose tissue via a peroxisome proliferator-activated receptor alpha- (PPARα-) dependent mechanism. PPARα is activated by increased free fatty acids in plasma following lipolysis [45]. In the current study, a stepwise regression analysis that also included BMI in the model identified WC and percentage body fat as the only independent determinants of circulating FGF-21 levels. While BMI reflects total adiposity, WC is a surrogate measure of abdominal adiposity [46] and to a larger extent reflects visceral fat content [47]. Our findings, therefore, implicate abdominal adiposity and increased visceral fat content as strong predictors of circulating FGF-21 in overweight and obese African males. This adds to the growing evidence that show visceral fat content as a superior determinant of circulating FGF-21 levels in populations with different ethnicities [17, 20, 21].

The importance of FGF-21 as a potential therapeutic agent and a diagnostic biomarker for metabolic diseases is increasingly being realized [2–9]. Administration of exogenous FGF-21 ameliorates several physiological perturbations related to metabolic diseases in animal models of obesity and diabetes (reviewed in [48]). Likewise in humans, FGF-21 analogs have shown beneficial effects on body weight and glucose control in obesity and diabetes [43, 49]. Despite beneficial effects, the paradoxical elevation in the circulating levels of this endocrine regulator observed in different disease states has raised concerns of whether a state of FGF-21 resistance exists in diseased humans [7, 11]. Equally, in the current study, levels of FGF-21 in the circulation were higher in T2DM when compared to nondiabetic subjects. These findings substantiate the previously reported FGF-21 paradox in an African population and further confirm a possible link between circulating FGF-21 and disturbed glucose and lipid metabolism.

The paradoxical elevation of FGF-21 levels in the circulation has also been reported in nondiabetic overweight and obese humans [10, 11, 15], indicating that the contentious regulation starts earlier than the actual time when a disease develops. This is partly attributed to an increase in adipose mass (adiposity), which usually precedes and triggers many cardiometabolic diseases (reviewed in [50]). Adipose tissue is a known source of circulating FGF-21, and any change in its quantity or quality may have a direct impact on FGF-21 in the circulation. In addition, perturbations in adipose biology are closely linked to impaired lipid profile in the circulation [51–53]. In this regard, FGF-21 regulation and biological effects may closely relate to lipid metabolism and its circulating profile. The association between circulating FGF 21 and lipid profile has been previously reported. In the current analysis, we also found a weak but significant association between circulating FGF 21 and lipid profile. Levels of circulating FGF-21 were positively correlated with total cholesterol, TG, and LDL, with HDL exhibiting a negative correlation. These associations however disappeared after adjusting for other covariates.

Collectively, the association between FGF-21, adiposity, and lipid profile indicates that FGF-21 can predict diseases that result from perturbed lipid metabolism. Certainly in longitudinal studies, high levels of circulating FGF-21 at baseline predicted the development of cardiometabolic diseases in humans [8, 54]. Interestingly, lifestyle factors such as diet and physical activity impact on circulating FGF-21, suggesting that levels of FGF-21 in the circulation can be used to monitor the effectiveness of these lifestyle factors [55, 56]. Given the reproducibility of findings in an African population evidenced herewith, FGF-21 therapeutic and diagnostic potential will possibly be evident in different African populations.

## 5. Conclusion

In a population of African males with or without type 2 diabetes, FGF-21 portrays a similar pattern in the circulation as previously reported in Asian and European populations. Levels are higher in diabetic than nondiabetic subjects and independently correlate with adiposity in overweight and obese subjects.

## 6. Study Limitation

The homogenous population (males only) used in this study increases the statistical strength, but also introduces limitations. The findings may not apply to female subjects. In addition the small sample size used, create further generalizability challenges. The interpretation of these findings is therefore limited to overweight and obese males. Given the importance of this metabolic regulator, further research to confirm its biological regulation in other African populations is mandatory.

## Figures and Tables

**Table 1:** Anthropometric and biochemical characteristics of the study population.

Variable	Disease status		
	Nondiabetic	T2DM	
Number of subjects enrolled	106	101	*p-value*
Weight, mean (SD), kg	77.7 (10)	80.6 (11.3)	0.045
Age, mean (SD), yrs	55.0 (6.3)	57.7 (5.9)	0.002
BMI, mean (SD), kg/m^2^	29.0 (2.9)	28.6 (3.3)	0.33
WC, mean (SD), cm	98.5 (10.0)	104.0 (10.1)	<0.001
Fat mass, mean (SD), kg	56.0 (7.2)	56.8 (7.9)	0.465
Percentage fat, mean (SD), %	27.7 (3.8)	29.4 (4.9)	0.007
Hypertension			
** **SBP, mean (SD), mmHg	133.8 (14.5)	138.4 (10.3)	0.009
** **DBP, mean (SD), mmHg	86.0 (7.9)	86.6 (8.3)	0.569
Glucose			
** **Fasting glucose, mean (SD), mmol/L	6.3 (0.5)	7.6 (1.8)	<0.001
Lipid profile			
** **Cholesterol	6.1 (1.1)	5.9 (1.2)	0.235
** **HDL, mean (SD), mmol/L	1.6 (0.4)	1.4 (0.5)	0.001
** **LDL, mean (SD), mmol/L	3.5 (0.8)	3.4 (0.9)	0.262
** **TG, mean (SD), mmol/L	1.6 (0.04)	2.3 (1.3)	<0.001
** **HbA1C, mean (SD), %	5.8 (0.6)	7.4 (1.6)	<0.001
FGF-21 mean (range) pg/ml	285.3 (122.9–811.0)	372.1 (100.0–810.6)	<0.001

SD = standard deviation.

**Table 2:** Spearman ranking correlation of serum FGF-21 levels with anthropometric and biochemical parameters.

Variable	All subjects		Nondiabetic		T2DM	
	r	*p*	*r*	*p*	*r*	*p*
Weight	0.402	<0.001	0.324	0.001	0.422	<0.001
Age	0.167	0.016	0.114	0.246	0.138	0.169
BMI	0.373	<0.001	0.403	<0.001	0.410	<0.001
Waist circumference	0.648	<0.001	0.507	<0.001	0.730	<0.001
Fat mass	0.020	0.772	0.024	0.809	−0.005	0.959
Percentage fat mass	0.770	<0.001	0.698	<0.001	0.799	<0.001
Systolic blood pressure	0.027	0.7	0.080	0.413	−0.131	0.193
Diastolic blood pressure	0.104	0.134	0.061	0.537	0.147	0.144
Fasting glucose	0.165	0.017	-0.037	0.705	0.094	0.349
Cholesterol	0.189	0.006	0.184	0.059	0.247	0.013
HDL	−0.355	<0.001	−0.164	0.093	−0.387	<0.001
LDL	0.154	0.026	0.145	0.137	0.198	0.047
TG	0.332	<0.001	0.265	0.006	0.326	0.001
HbA1C	0.226	0.001	−0.021	0.831	0.271	0.006

**Table 3:** Stepwise multiple regressions of variables associated with the serum level of FGF-21.

Independent variables	*β*	*t*	*p-value*	95% CI
Age (years)	0.392	0.39	0.697	(−1.594 to 2.379)
Fat mass (%)	18.019	11.07	<0.001	(14.808–21.223)
Waist circumference	4.608	5.68	<0.001	(3.008–6.206)
Uiuml	2.472	3.52	0.001	(1.086–3.859)
TG	13.396	2.14	0.033	(1.061–25.732)
HOM-air	−4.949	−2.38	0.018	(−9.044 to −0.853)
HbA1c	10.36595	1.93	0.055	(−0.205 to 20.936)
BMI	−3.151958	−1.37	0.171	(−7.677 to 1.373)
Adjusted *R*^*2*^	0.69			

BMI = body mass index. CI = confidence intervals.

## Data Availability

The data used to support the findings of this study are available from the corresponding author upon request.
